# Acknowledgement to Reviewers of *Foods* in 2015

**DOI:** 10.3390/foods5010009

**Published:** 2016-01-22

**Authors:** Foods Editorial Office

**Affiliations:** MDPI AG, Klybeckstrasse 64, CH-4057 Basel, Switzerland; foods@mdpi.com

The editors of *Foods* would like to express their sincere gratitude to the following reviewers for assessing manuscripts in 2015. 

We greatly appreciate the contribution of expert reviewers, which is crucial to the journal’s editorial decision-making process. Several steps have been taken in 2015 to thank and acknowledge reviewers. Good, timely reviews are rewarded with a discount off their next MDPI publication. By creating an account on the submission system, reviewers can access details of their past reviews, see the comments of other reviewers, and download a letter of acknowledgement for their records. In addition, MDPI has launched a collaboration with Publons, a website that seeks to publicly acknowledge reviewers on a per journal basis. This is all done, of course, within the constraints of reviewer confidentiality. Feedback from reviewers shows that most see their task as a voluntary and mostly unseen work in service to the scientific community. We are grateful to our reviewers for the contribution they make.

Aguedo, MarioCavazza, AntonellaFitton, HelenAlavi, SajidChang, Ching-JuiFjeld, HeidiAllegaert, KarelChang, Fang-RongFletcher, Paul C.Aluko, RotimiChaves-Lopez, ClemenciaFontecha, JavierArvanitoyannis, IoannisChen, HuaiqiongFortunati, ElenaAubourg, Santiago P.Chiang, Wen-DeeFoster, Jane A.Barba, Francisco J.Ciani, MaurizioFraqueza, Maria JoãoBeasley, SheaDas, UndurtiFrígola, AnaBeatty, StephenDonno, Dario GonzÁlez-DÍaz, HumbertoBermúdez-Aguirre, DanielaDreij, KristianGonzález-Mas, M. CarmenBianco, Ismael D.Drosinos, Eleftherios H.Greco, LuigiBidwell, JosephDurango, Diego L.Gruszecki, WieslawBlanco Penedo, IsabelDurazzo, AlessandraGuerzoni, Maria ElisabettaBourquin, LeslieEconomou, VangelisHallmann, EwelinaBritten, MichelEdwards, SandraHamdy Roby, Mohamed HusseinBurrows, Tracy L.Elansary, Hosam O.Hansen, Tina BeckCanavari, MaurizioElkhalifa, Abd Elmoneim O.Harrold, Joanne A.Cano, AmaliaEverett, David W.Hasanuzzaman, MirzaCaporaso, NicolaFabra Rovira, M JoseHenriques, Marta H. F.Cardoso, MarisaFåk, FridaHolck, Askild LorentzCasal, SusanaFeigl, BeatrixHolley, Richard A.Castro, SoniaFerragut, VictoriaHönow, RuthHospital, Xavier F.Masson, Lourdes M. P.Rocha Guzmán, Nuria ElizabethHsu, Chia-LinMataragas, MariosRodriguez-Mateos, Ana M.Iametti, StefaniaMattei, JosiemerRosa Loizzo, MonicaIbrahim, Salam A. Miller, CharlesRumpold, BirgitIossifidou, EleniMiyagi, AtsukoSalafranca Lázaro, Francisco J.Jahncke, MichaelMolenaar, JaapSantillo, AntonellaJensen, Morten GeorgMorabito, AntoninoSantini, AntonelloJohansson, EvaMoreira , Ramón Scherf, KatharinaJohnson-Kozlow, MarilynMoreno, SilviaSgroi, FilippoJordan, KieranMorita, KyojiSheikh, Ali AhmadJoseph, LucyMu, Jianhong E.Silva, Juan L.Juneja, VijayMuñoz-Alférez, M. JoséSmith, BrennanJuszczak, LesławMyers, JamesSobarzo-Sánchez, EduardoKasarda, DonNag, ArupSoultos, NikolaosKhachik, FredNemeghaire, StéphanieSpeer, KarlKimiya, TakashiNewnham, EvanSu, Marcia Shu-WeiKjarnes, UnniNicolai, TacoSyed, MoinuddinKnight, JohnNicoli, Maria CristinaTalens, PauKojo, ShosukeNiewold, TheoTarantino, GiovanniKomaitis, MichaelNolan, JohnTassou, Chrysoula C.Koppel, KadriNovellino, EttoreTopp, KairstyKourkoutas, YiannisOmer, SayedTormod, MørkKovbasnjuk, OlgaPanagou, EfstathiosTorrieri, ElenaLalas, StavrosPark, Chung GyooTzia, ConstantinaLamuela Raventós, Rosa MaríaParsons, Barry J.Ueno, HidetoLangton, MaudPérez-Lamela, ConcepciónVelickova, ElenaLarre, ColettePerrone, SerafinaVerhagen, HansLeone, AntonellaPiga, AntonioVermeer, Herman MaartenLillioja, StephenPlourde, GuyErdman, John W. Lo Scalzo, RobertoPrakash, AnuradhaWallace, R. JohnLongo, VincenzoPuppo, CeciliaWang, Chung-YiLoveday, Simon M.Rajagopal, S. N.West, GaleMaher, PamelaRallabhandi, PrasadWu, Chi-ReiMaheshwari, AkhilRembialkowska, EwaXin, Xiu-FangMarchello, JohnRivas, AnaYamamoto, KazutakaMarín, SoniaRivero-Pérez, M. DoloresYing, DanyangMartinez-Monteagudo, SergioRobitaille, GillesZhu, Yefei

